# Factors that positively influence adherence to antiretroviral therapy by HIV and/or AIDS patients and their caregivers

**DOI:** 10.4102/phcfm.v3i1.196

**Published:** 2011-08-04

**Authors:** Andrew J. Ross, Myint Aung, Laura Campbell, Gboyega A. Ogunbanjo

**Affiliations:** 1 Department of Family Medicine, University of KwaZulu-Natal, South Africa; 2 Dundee Hospital, KwaZulu- Natal, South Africa; 3 Department of Family Medicine and Primary Health Care, University of Limpopo (Medunsa Campus), South Africa

## Abstract

**Background:**

The importance of dedicated adherence to antiretroviral treatment (ART) in the management of Human Immunodeficiency Virus (HIV) is well documented. Multiple factors may affect adherence and this study explores patients’ and their caregivers’ perceptions of factors which may positively influence adherence to ART.

**Method:**

This study was a descriptive, qualitative study that used both free attitude interviews and focus-group discussions. Nineteen patients attending a busy ART-clinic at a district hospital in KwaZulu-Natal and eight caregivers were purposefully selected. Selection criteria included good adherence to ARTs as evidenced by excellent clinic attendance for more than one year with evidence of clinical, immunological and viral improvement. Interviews were tape recorded, transcribed and thematically analysed.

**Results:**

Ten female participants, nine male participants and eight caregivers took part in the study. Participants highlighted three main categories that positively affect their adherence to ART namely: patient, disease and health care provider-related factors. Sub-themes included issues related to acceptance, disclosure to significant others, symptomatic improvement on ARTs and the importance of supportive relationships. Participants greatly valued the health care provider relationship and felt that the main role of the health care provider was to educate and support.

**Conclusion:**

This study has shown that the factors which most influenced adherence were patient-related (acceptance, disclosure, determination, and family support), disease-related and treatment-related (symptomatic illness and improvement on ARTs), and healthcare worker-related (relationships, and adherence classes).

## Introduction

### Setting

Antiretroviral treatment (ART) has changed Human Immunodeficiency Virus (HIV) infection from a fatal disease to a chronic illness which can be managed much like other chronic illness.^1^ The importance of high levels of adherence to ART in the management of HIV infection is well documented^2, 3^ and are critical in preventing the development of viral resistance and subsequent immunological and clinical failure of ART.^4^ Multiple issues have been identified which may affect adherence, including patient factors (depression, substance abuse, treatment beliefs), treatment factors (regimen complexity, side effects), and contextual factors.^5^

A meta-analysis of 21 African studies carried out in 2006 showed that 77% of 12 116 HIV positive patients had adherence rates of 95% to ART.^6^ A Tanzanian study conducted in 2009, demonstrated that adherence was related to the level of trust between the patient and the health care provider, however this relationship was not considered to be a major factor affecting adherence in other studies.^7^ Other factors that were considered important for adherence were:

- improvement in health after starting ART
- perceived need to meet family responsibilities
- development of routines
- emotional support received from others.^8^

There is a paucity of data on patients’ perceptions of factors associated with good adherence in KwaZulu-Natal, South Africa, which has the largest prevalence of HIV disease in the world.^9^ The aim of this study was to explore, from the patient and caregiver perspective, factors that positively influence adherence to ART. This study was based at a district-level public hospital in KwaZulu-Natal serving both urban and rural populations. The study site was accredited as an ART roll-out site in 2004, and by 2005 there were 542 patients on the ART programme.

## Ethical considerations

Ethical permission was obtained from the University of KwaZulu-Natal, Ethics Committee and the Department of Health, KwaZulu-Natal.

## Methods

This was a descriptive, qualitative study using free attitude interviews (FAI) and focus group discussions (FGD). The study population was all patients attending the ART clinic older than 18 years of age adherent to ART. Twenty one patients were purposively selected by the researcher to participate in the study. Patient selection criteria included: ability to speak English as the researcher was English speaking, willingness to participate in the study, attendance of the ART clinic for at least one year and good adherence to ART defined as:

- undetectable viral load
- increasing CD4 count
- no missed clinic visits
- pill count indicating over 95% compliance
- symptomatic improvement.

All the interviews were conducted in English by the principal researcher and this is noted as a study limitation because the study was conducted in English. Signed consent was obtained from each participant prior to the interviews and discussions. A single exploratory question was asked: ‘*What enables you to achieve good adherence to your ART medication?’* Interviews were facilitated by the principal researcher and field notes were kept. Four FAIs and two FGDs were conducted at the hospital. Two FGDs were conducted in patients` homes with family members in an attempt to overcome any potential bias created by conducting interviews in the hospital environment and to obtain the opinion of caregivers on factors promoting adherence to ARTs. All interviews were audio-taped and transcribed to text. The transcripts were presented to the participants to ensure content validity and were then coded thematically, by a team of experienced researchers. Triangulation was achieved by audiotaping the interviews, compiling field notes to ensure confirmation and completeness of the data and by member checks of the data by the participants.

## Results

Nineteen patients and eight caregivers (family members) participated in the study. Two of the patients selected did not participate in the FGDs; one women excused herself on the day of the interview and one man arrived too late to participate. Data saturation was reached by the end of these interviews.

Three major categories of factors identified as positively influencing adherence were:

- patient-related factors
- disease-related and treatment-related factors and
- healthcare-related factors.

A summary of those who participated in the FGDs and FAIs is presented in Table 1 and a summary of the themes identified is presented in Table 2. Although themes have been identified under each heading, this division of the themes is artificial. Adherence is multifactorial and the themes are interlinked e.g. clinical improvement helps patients to accept the diagnosis and the need to be adherent to medication, disclosure allows for support and support in turn facilitates disclosure. Patient-related factors were further sub-themed into five themes, disease-related and treatment-related factors into two themes and healthcare-related factors into two themes.

**TABLE 1:** Participants in the focus group discussions (FGDs) and free attitude interviews (FAIs).

FGDs and FAIs	Patients		Family members		Venue	Total number
	
	Male	Female	Male	Female		
FDG 1	-	7	-	-	Hospital	**7**
FDG 2	6	-	-	-	Hospital	**6**
FDG 4	1	-	1	2	Patient's home	**4**
4 FAIs	2	2	-	-	Hospital	**4**

*Source*: Authors’ original data

FGD, focus group discussion; FAI, free attitude interview.

**TABLE 2:** Themes and subthemes identified which influence adherence.

Themes	FGD				FAI				Total
	
	Hospital		Homes		Female		Male		
			
	Male	Female	Male	Female	1st interview	2nd interview	1st interview	2nd interview	
**Patient-related factors**									
Acceptance of HIV status	√	√	√	√	√	√	√	√	**8**
Previous experience	√	√	√	√	√	√	√	√	**8**
Belief in ART	√	√	0	√	√	0	0	0	**4**
Disclosure to significant others	√	√	√	√	√	√	√	√	**8**
Personal and family responsibility	√	0	√	√	√	√	√	0	**6**
**Disease-related and treatment-related factors**									
Symptomatic	√	√	0	0	√	√	√	√	**6**
Improvement on ARTs	√	√	0	0	√	0	0	√	**4**
**Healthcare-related factors**									
Supportive relationships	√	√	√	0	√	0	√	√	**6**
Adherence classes	√	√	√	√	0	√	√	0	**6**

*Source*: Authors’ original data

FGD, focus group discussion; FAI, free attitude interview; HIV, human immunodeficiency virus; ART, antiretroviral therapy.

### Patient-related factors

The following patient-related factors were identified as having played an important role in promoting good adherence to ARTs.

#### Acceptance of HIV status

Patients acknowledged that they had to ‘accept [my HIV] status’ and ‘take it as a disease and accept it positively’ if they were to be adherent to ARTs. A patient considered that ‘some are not doing well because they don't accept their status [sic]’. Patients acknowledged that it is essential for them to come to terms with the fact that ‘I am HIV positive and I need to take medication for the rest of my life’ and for them to be adherent to ARTs.

#### Previous experience

Good adherence was enhanced by previous experiences. These experiences where both positive and negative and included *‘learn*[*ing*] *about HIV from* [*my*] *late son’* which motivated Mrs BT to encourage her son to take his ART because ‘*we do not want to lose another one*’. Ms. KMN experience with traditional medication on which *‘I became thinner and thinner’* led her to conclude that *‘only ART is helping me get better’*. This sentiment was echoed by Ms. KT who said *‘When I used to take immune booster tablets I didn't get better but when I'm taking ART my weight goes up. I trust ART*, *100%’*.

#### Belief in antiretroviral treatments

Belief that HIV must be managed with ARTs and not with alternatives such as traditional medication is an essential component of good adherence. *‘I never use traditional medicine, ART is number one’* and *‘I believed in* [*Western*] *medicine so that I went to hospital. I don't believe in traditional medicine’ w*as the sentiment expressed by a number of patients.

#### Disclosure to significant others

Disclosure to family members and friends was seen as essential to successful adherence. Even though ‘*it is difficult to disclose*’, all participants were of the opinion that *‘disclosure is very important’* and that *‘we must disclose to our relatives and family’*. Disclosure allowed for support which played a vital role in encouraging good adherence. The following comments from participants indicated the huge impact that informed families had on ensuring compliance in very practical ways such as:

- ‘reminding me … they wake me up for pills’
- ‘telling me, you have to be strong’
- ‘cooking healthy food’
- ‘supporting me when I was so sick’
- ‘paying money for me to go to hospital for attending classes’.

Disclosure allows for a treatment supporter who ‘helped me a lot (by) reminding me of my treatment, visiting and encouraged me’ and support from others, ‘My boss encouraged me a lot. She took me to VCT with her own car. She gave me a lot of support like food parcels and encouraged to go for VCT’.

Patients contrasted disclosure with non-disclosure which they felt impacted negatively on patients’ ability to take their medication. Participants felt that if ‘*… patients hide their status and their ARV treatment’* because they are *‘… shy to take medication in the presence of other people and hide their treatment’* and if they *‘do not accept* [*their*] *HIV status’* and continue to *‘… tell you that they have TB, not HIV, because they denied it’* they are unlikely to adhere to ART medication.

#### Personal and family responsibility

Personal responsibility and accountability were identified as key factors in ensuring adherence to ARTs. Participants acknowledged that: *‘I must take responsibility for my life’* because *‘I want to live longer’*. Mr SJM expressed it as *‘my life is… important, I must take care of my life by myself’*.

Personal responsibility extended to life style changes necessary to promote health. These included:

- ‘eating healthy food before taking ARVs’
- ‘I wake up early and go jogging’
- ‘not using alcohol, and not smoking’
- ‘never blaming each other, we started safe sex. If I forget, she reminds me for condom’.

A sense of responsibility to their family was a strong motivating factor for many patients to be adherent to ARTs. Ms MZ stated that *‘I don't want to die because I need to raise my family. I'm the breadwinner’*.

Inadequate financial resources were seen to undermine good adherence to ARTs. This was clearly expressed as ‘no job, no support, very difficult to get good adherence’ and ‘no money to eat a decent meal in order to take the tablets. If [patients] take the tablets on an empty stomach they feel sick’.

### Disease-related and treatment-related factors

#### Being symptomatic and improving on antiretroviral treatments

Ms KMN remembered when she was ‘*… so sick, I could not walk myself. My daughter used a wheelchair to bring me to the clinic. Now, I'm very happy with ART’*. Being symptomatic and improving on ARTs was a strong motivating factor for patients to take their ARTs ‘*… because I want to live’*.

Good adherence was rewarded and re enforced because ‘there is improvement in my life with ART’ which gave patients ‘a second chance of life through ART’. Participants were able to report that ‘I was so thin after giving birth, but look at me now’ and ‘I am gaining weight and have come back to normal life like before I was sick’. This improvement enabled many patients to return to work and provide for their children.

Participants recognised that ‘*the tablets are many’* and that *‘the side effects are there’*. However, adherence training had prepared them and did not impact on the patients’ determination to take ARTs as *‘the side effects do not exceed the power* [*determination*] *to be alive’*.

### Healthcare-related factors

#### Supportive relationships with health care providers

Establishing a supportive relationship between Health Care Providers (HCPs) who were knowledgeable, available, caring, empathetic, ‘*helpful and approachable’* and patients was identified as very important in enabling patients to achieve high levels of adherence to ART. Patients recognised the vital role played by staff members in supporting them and providing continuous counselling. This supportive relationship meant that *‘when we have problems or side effects like vomiting we can consult our doctor’* as *‘they are so patient for HIV-*[*infected*] [*sic*] *patients; they encourage me to take treatment properly’*.

#### Adherence classes

Participants felt that adherence counselling was extremely helpful in ensuring good adherence. Ms KMN stated ‘*I'm taking medicine very well because I clearly understand the disease*’. Adherence counselling allows patients to be ‘*educated and misconceptions corrected*.’ During adherence classes ‘*they explained that the drug can help but not cure; I need to take treatment properly and I have to take them for the whole of my life*’ and that ‘*lessons helped me to be positive and do well with my medication*’. The literacy training also ‘*helped a lot and we learnt how to take medicine, what the side effects are, and healthy life style*’. Patients were even able to say that *‘if you have side effect, please seek help from the doctor, do not stop ART’* because *‘ART makes me different. I could die if there was no ART’*.

Literacy training also plays a vital role in reducing stigma and improving family support. Mrs BT, who attended the training with her daughter, indicated that literacy enabled them to understand more about HIV through their combined knowledge. As a result ‘*we did not separate her from our family. We even use same utensils together* [*because*] *we were not scared of HIV*‘.

## Discussion

With 750 000 patients on ARTs in South Africa and with more than 95% adherence needed to sustain viral suppression and immune recovery,^4^ it is essential that HCPs learn as much as possible about factors which promote adherence to ART.

The Oxford Concise Medical Dictionary has simplistically defined adherence as *‘the degree to which a patient correctly follows medical advice’*.^10^ The determinants of adherence are however extremely complex, involving internal factors such as beliefs, knowledge, determination, the perceived seriousness of the illness, the efficacy of treatment as well as external factors such as financial cost, accessibility, support and advice.^11^

Many studies have identified factors influencing adherence to ARTs. Most have focused on issues of non-adherence and have divided these into:^4, 5, 8, 12^

- patient-related factors (age, gender, depression, substance abuse, disclosure and non-disclosure, support, finances, fear of stigmatisation)
- disease-related factors (symptomatic)
- treatment-related factors (side effects of medication, complexity of treatment regime)
- the patient-healthcare-provider relationship
- health-system-related factors (accessibility, staffing levels).

It is interesting to note that the majority of the issues identified in this study as important, are issues relating to good adherence.

Belief and acceptance are necessary starting points for adherence, re-enforced by a previous (often negative) experience, and symptomatic improvement and knowledge provided during adherence classes. Although these are not new issues and have been identified in previous research conducted in Tanzania, Cote d'Ivoire and South Africa,^2, 8, 13^ it is essential that HCPs are aware of the role that each of these factors play and use them to promote adherence. If patients have relatives who have died from AIDS, this could be used to motivate them to take their medication. If patients have taken traditional medication previously and have not improved, this too can be used to re-enforce the need to be adherent to ARTs. Disclosure to significant others appeared to be a key factor in promoting adherence and was identified by patients as critical for good adherence. Patients were of the opinion that without disclosure there could be no support. Disclosure allowed families to be involved in the care and support of their loved one. Watt reported that disclosure to family members promoted adherence in practical ways such as reminders to take pills, and material support such as the provision of financial support and food.^8^ The importance of disclosure has been recognized in other studies and is a pre-requisite for patients accessing ARTs at South African government roll-out sites.^2, 3, 5^) A study conducted in Tanzania found that ‘tangible and emotional support from family members facilitated adherence’.^8^ In contract, a study from Cote d'Ivoire identified absence of social and family support as a factor leading to poor adherence.^13^

HIV affects individuals at the height of their reproductive lives and many of those who are taking ARTs have family members who depend upon them for material and financial support. This study identified family responsibilities as an important motivating factor promoting adherence and can be used by HCPs as a means of motivating adherence. This finding is similar to that of Watt in Tanzania and Aspeling in South Africa who demonstrated that family responsibility and the desire to raise one's own child were important motivating factors in promoting adherence.^8, 14^

In keeping with the finding of Watt in Northern Tanzania,^8^ patients in this study were able to connect the symptoms that resulted from their HIV-infection, an improvement in health and their adherence, to ARTs. An important role of HCPs is to help patients to link symptomatic improvement to adherence and HCPs must continually reinforce this connection and use it as a motivating factor when encouraging patients to be adherent to ARTs.

Adherence classes are essential as they provide information about the disease, its progression, the correct doses of medication, and possible side effects: all of which are important for patients and help to ensure adherence to medication. This is consistent with the findings of other studies.^8^

If access to a team of knowledgeable, sympathetic and understanding patient-centered HCPs is available, patient- adherence to ARTs is enhanced. This therapeutic alliance has been found by Aspeling, Watt and others to be very important and should not be underestimated.^8, 14, 15^ McWhinney states that the doctor-patient relationship is an essential component in managing any chronic illness^16^ and Vermeire, Hearnshaw and others state that ‘one of the most commonly advocated ways to improve compliance is the improvement of the doctor- patient relationship’.^17^ A key challenge in this relationship is to build a therapeutic alliance, where the role of both parties is recognised and the patient and HCPs work together to ensure that the patient's illness is managed optimally. In the area of HIV-care this alliance not only is with the doctor but with a team of health care professionals, who provides care for the patient. This ideal however, is difficult to achieve and to sustain in a health care system that is grossly underfunded, massively overburdened and where staff morale is extremely low. Despite these challenges, HCPs need to be aware that their patients value them greatly and that they have a vital and positive role in optimizing adherence to ART.

### Limitations

The study was conducted in English at only one site in KwaZulu-Natal.

## Conclusion

This study has shown that the factors which influenced adherence the most were patient-related (acceptance, disclosure, determination, and family support), disease- related and treatment-related (symptomatic illness and improvement on ARTs), and healthcare worker-related (relationships, and adherence classes).

The study confirmed from the patient's perspective what other studies have shown: that acceptance of the disease is crucial, and disclosure is paramount. It is the gateway to essential support. Adherence classes are critical in improving knowledge, understanding, preparation for dealing with side effects and the long duration of treatment and in decreasing stigma. HCPs need to be aware of the factors which promote adherence and to recognise the crucial role they play in supporting, encouraging and walking with patients on their adherence journey.
